# Comparative effectiveness of Biobrane®, RECELL® Autologous skin Cell suspension and Silver dressings in partial thickness paediatric burns: BRACS randomised trial protocol

**DOI:** 10.1186/s41038-019-0165-0

**Published:** 2019-10-31

**Authors:** Anjana Bairagi, Bronwyn Griffin, Zephanie Tyack, Dimitrios Vagenas, Steven M. McPhail, Roy Kimble

**Affiliations:** 1 Centre for Children’s Burns and Trauma Research, Centre for Children’s Health Research, Brisbane, Queensland, Australia; 2 Pegg Leditschke Children’s Burns Centre, Queensland Children’s Hospital, Brisbane, Queensland, Australia; 3 School of Nursing, Institute of Health and Biomedical Innovation, Queensland University of Technology, Brisbane, Queensland, Australia; 4 Australian Centre for Health Services Innovation, Institute of Health and Biomedical Innovation, School of Public Health and Social Work, Queensland University of Technology, Brisbane, Queensland, Australia; 5 Research Methods Group, Institute of Health and Biomedical Innovation, Queensland University of Technology, Brisbane, Queensland, Australia; 6 The University of Queensland, Brisbane, Queensland, Australia; 7 Centre for Functioning and Health Research, Metro South Hospital and Health Service, Brisbane, Queensland, Australia

**Keywords:** Partial thickness burns, Children, Regenerative epidermal suspension, Re-epithelialisation, Wound healing

## Abstract

**Background:**

Mixed partial thickness burns are the most common depth of burn injury managed at a large Australian paediatric hospital specialty burns unit. Prolonged time until re-epithelialisation is associated with increased burn depth and scar formation. Whilst current wound management approaches have benefits such as anti-microbial cover, these are not without inherent limitations including multiple dressing changes. The Biobrane® RECELL® Autologous skin Cell suspension and Silver dressings (BRACS) trial aims to identify the most effective wound management approach for mixed partial thickness injuries in children.

**Methods:**

All children presenting with an acute burn injury to the study site will be screened for eligibility. This is a single-centre, three-arm, parallel group, randomised trial. Children younger than 16 years, with burns ≥ 5% total body surface area involving any anatomical location, up to 48 h after the burn injury, and of a superficial partial to mid-dermal depth, will be included. A sample size of 84 participants will be randomised to standard silver dressing or a Regenerative Epithelial Suspension (RES™) with Biobrane® or Biobrane® alone. The first dressing will be applied under general anaesthesia and subsequent dressings will be changed every 3 to 5 days until the wound is ≥ 95% re-epithelialised, with re-epithelialisation time the primary outcome. Secondary outcomes of acute pain, acute itch, scar severity, health-related quality of life, treatment satisfaction, dressing application ease and healthcare resource use will be assessed at each dressing change and 3, 6 and 12 months post-burn injury.

**Discussion:**

The findings of this study can potentially change the wound management approach for superficial partial to mid-dermal burns in children locally and worldwide.

**Trial registration:**

The Australian New Zealand Clinical Trials Registry (ACTRN12618000245291) approved prospective registration on 15 February 2018. Registration details can be viewed at https://www.anzctr.org.au/Trial/Registration/TrialReview.aspx?id=374272&isReview=true.

## Background

Burns are the fifth most common cause of non-fatal injuries in children younger than 16 years [1]. Paediatric burn injuries are associated with the burden of both functional and psychological challenges to the child [2–4] as well as the considerable expense incurred to health service providers who manage the burn care [5]. In 2017, three-fourths of acute burns treated at the Queensland Children’s Hospital in Brisbane and approximately half of recorded burn cases across Australia and New Zealand [6] were partial thickness burn injuries. The available wound management options are not without inherent disadvantages including but not limited to delayed wound healing [7–12]. Thus, current practice is driven to facilitate faster wound re-epithelialisation to improve scar outcomes [13] and reduce healthcare costs [10, 14].

Partial thickness burn injuries have a varied time to re-epithelialisation. Superficial partial thickness burns ideally heal within 14 days without requiring surgical intervention. Typically, deep partial thickness wounds are anticipated to have a prolonged time to re-epithelialisation (TTRE), often requiring a skin graft. Mid-dermal thickness wounds can potentially re-epithelialise spontaneously, within 14 days and without surgical intervention [15]. However, this is not always the status quo and management of intermediate depth burns remains contentious. Some surgeons prefer early skin grafting, whereas others advocate for conservative non-surgical management.

One third of paediatric burns will develop hypertrophic scar, if wound re-epithelialisation occurs between 14 and 21 days [15–17]. It is presumed that children have an increased collagen production rate hence the higher incidence of hypertrophic scar formation as compared with adults [18]. In a systematic review, Vloemans et al. found that membranous dressings were better than topical standard of care options for the treatment of partial thickness scald burns in children [19]. However, there was no clear recommendation regarding the best membranous dressing for treatment of partial thickness paediatric burns [16]. Burn depth, TTRE and hypertrophic scar have an established association [13, 15–17]. Yet, despite advances in wound management approaches, the ideal dressing to manage partial thickness burns in children remains undefined. The three wound management approaches to be investigated in this trial are a topical anti-microbial dressing, epidermal replacement using an autologous skin cell suspension and dermal salvage using a biosynthetic skin substitute.

Topical silver impregnated dressings used as the standard of care at the study site are (i) Acticoat® (Smith and Nephew, Hull, UK) with Mepitel® (Mölnlycke, Göteborg, Sweden) or (ii) Mepilex Ag® (Mölnlycke, Göteborg, Sweden) alone [9]. Acticoat®, is a multi-layered polyethylene nanocrystalline silver pad that is attached to a soaking coat of polyester [20] and has been in clinical use since 1993. When compared to silver sulphadiazine ointment, Acticoat® is safe, cost-effective and reduces the TTRE, requirement for grafting and long term scar management in paediatric burn injuries [21–23] . Mepitel® is a silicone-coated, nylon dressing with a silicone wound interface layer. Acticoat® combined with Mepitel deliver antimicrobial properties of silver nanocrystalline particles and an atraumatic pain-free wound contact layer. This promotes faster re-epithelialisation by minimising wound-related trauma and pain [24].

Mepilex Ag® is a silver sulfate soft silicone foam dressing that allows for continuous silver delivery to the wound, good exudate management, provides a moist environment and thermal insulation making this dressing ideal for partial thickness burns [25]. In a randomised trial of children (*n* = 96) with partial thickness burns, silver-impregnated foam dressings were effective in shortening the TTRE and reduced pain during dressing changes [9]. Despite this, silver impregnated dressings are more expensive per dressing change when compared to Biobrane® and a Regenerative Epithelial Suspension (RES™) [10], require frequent dressing changes due to non-transparent design and are associate with adverse effects such as cytotoxicity and argyria. Over the last 30 years, epithelial suspension preparation for burn wound management has undergone a multitude of transformations including the number of enzymatic degradation agents used, co-delivery systems with various cell carriers and a range of preparation times (few weeks to under an hour) [7, 26]. The RECELL® Autologous Cell Harvesting (ACH) device (Avita Medical, California, USA) was first introduced almost two decades ago [27]. In approximately 30 min, a Regenerative Epithelial Suspension (RES™) containing basal keratinocytes, melanocytes, fibroblasts, melanocytes, Langerhans cells and epidermal basal cells [28] is obtained from a donor skin sample as small as 1 cm^2^. The faster preparation allows for a readily available suspension compared to the cultured keratinocytes, where wait times can be a few weeks.

Few studies have comprehensively assessed the effectiveness of the RES™ in the management of childhood burn injuries. Most of the published literature is based on the adult cohort and there is a paucity of data based on the paediatric population. When compared to standard care, wounds treated with RES™ had longer operative times [29], more wound infection [10] and once applied the RES™ requires a retention dressing such as Biobrane®, to keep *in situ* [10]. However, in the other studies, application of RES™ was associated with less post-operative pain [10, 29], smaller donor site area [29, 30], shorter TTRE [10, 29, 30], fewer dressing changes and cost-effectiveness [10, 31]. Autologous skin cell suspensions show promising potential to change current burn wound management approaches from dressing based to cell-based therapy.

First introduced in 1979 [11], Biobrane® (Smith and Nephew, Hull, UK) is a composite biosynthetic skin substitute composed a matrix of short porcine collagen peptides bonded to a layer of nylon that is enveloped in silicone [8]. The benefits of using Biobrane® include shorter TTRE, improved mobility, shorter hospital stay, ease of application, ability to visualise the wound [32] and reduced pain [10–12, 33, 34]. For clean wounds, this transparent dressing is able to stay *in situ* for up to 14 days, thus supporting the cost-effective treatment of burn injuries [35]. Failure of Biobrane® to adhere to the wound bed occurs once counts exceed 10^5^ organisms/gram of tissue [36]. Reported infection rates associated with Biobrane® range from 5 to 22% [12, 37, 38]. However, Lal. et al. demonstrated that infection rate upon application of Biobrane® to superficial partial thickness scald burns in children with 48 h of injury did not differ when compared to a topical silver dressing [39]. Biobrane® recreates the barrier function of the skin and has been used as a dermal salvage option for burn wounds over the last four decades.

### Aims

The Biobrane®, RECELL® Autologous skin Cell suspension and Silver dressings Trial (BRACS Trial), will evaluate partial thickness, paediatric and burn injuries with the following aims:

#### Primary

To evaluate the effectiveness of three wound management approaches (standard care silver dressings (Acticoat® and Mepitel® or Mepilex Ag®)) or an autologous skin cell suspension (ASCS, harvested with the RECELL® ACH device) with Biobrane® or Biobrane® alone, in reducing the re-epithelialisation time of superficial partial to mid-dermal thickness burn injuries in children.

#### Secondary

To examine the effectiveness of three wound management approaches in the same group of children for reducing pain, itch, scar severity and healthcare resource use as well as, improving health-related quality of life, treatment satisfaction and dressing application ease.

### Study design

The BRACS Trial is a parallel group, single-centre and randomised trial. The null hypothesis is that there is no difference between the three intervention groups. The alternative hypothesis is that there is a statistically significant difference between the interventions. If null hypothesis is not rejected, this is still important, as treating clinicians will be able to select an intervention knowing that patient well-being will not be compromised. An active treatment control group (group A: standard silver dressings) was included as it was considered unethical to use a placebo group for this study.

## Methods

### Study setting

This single-centre study will be conducted at a major paediatric burn centre in Brisbane, Queensland, Australia. This large state hospital has a catchment area spanning more than 1.73 million km^2^ and with a population of approximately 5 million inhabitants [40]. The centre treats over 1200 new burns patients annually.

### Eligibility criteria

**Fig. 1:**
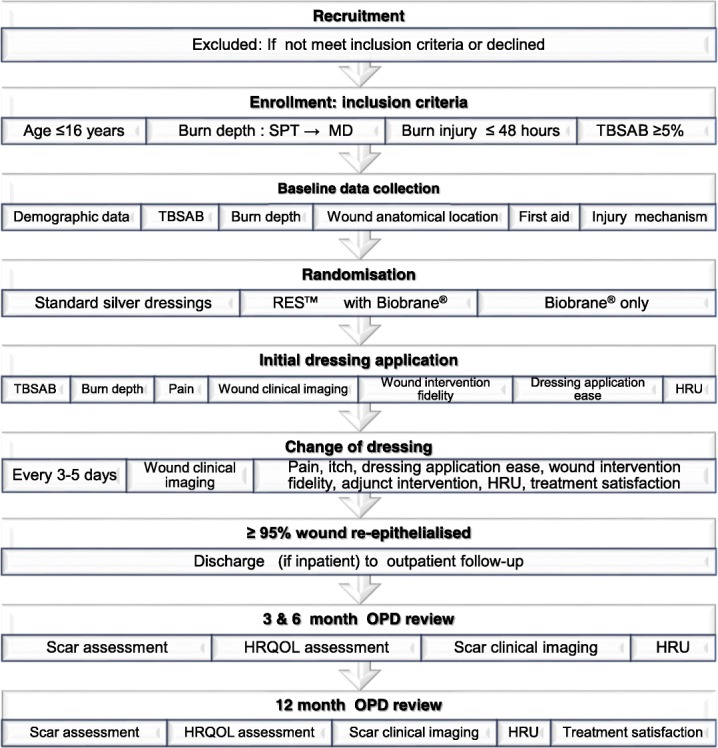
Biobrane^®^, RECELL® Autologous skin Cell suspension and Silver dressings (BRACS) Trial flow diagram. *SPT* superficial partial thickness, *MD* mid-dermal, TBSAB total body surface area burned, *RES™* Regenerative Epithelial Suspension, *HRU* Healthcare Resource Use, *HRQOL* Health-related quality of life, *OPD* outpatient department

All new patients attending the study site will be evaluated for eligibility. Children younger than 16 years, who have sustained a burn of superficial partial thickness to mid-dermal depth, within 48 h of injury and with a total body surface area burned (TBSAB) ≥ 5% as assessed by the attending burns surgeon, will be included. To allow for patients who travel from regional referring centres to be included, application of silver-impregnated dressings onto wounds prior transfer to the study setting will be included. Patients with superficial burns, deep dermal to full thickness depth wounds and injuries deemed not compatible with life by the attending burns surgeon, will be excluded from participation in the study, see Fig. 1.

### Recruitment

Treating clinicians of all children presenting to the study setting will determine eligibility for enrolment in the study based on the set inclusion and exclusion criteria. Participant (where applicable) ‘assent’ is sought at the recruitment of each potential participant. Once guardian permission is confirmed, informed consent for participation will be obtained by an investigator aligned with the study. A translator will be present for all participants of non-English speaking background throughout the study. All participants and their guardians will be given an opportunity to discuss the study with the investigator prior to signed consent. Participants, who decline enrolment into the study, will be assigned standard care and only their de-identified data will be collected.

### Interventions

Eligible participants will be randomised to one of the three interventions:

- Group A: standard silver dressings. Silver dressings will be applied ((i) Acticoat® with Mepitel® or (ii) Mepilex Ag®) as per standard protocol at the study site.
- Group B: Biobrane® combined with RES™
- Group C: Biobrane® only.

### Operative procedures

The initial dressing application will be completed under a general anaesthesia for all consented participants under aseptic conditions. The operative timeline is illustrated in Table 1. For eligibility to enrol, the attending burns surgeon will determine TBSAB and this will be the primary approach for TBSAB assessment. The inaccuracy of human TBSAB assessment arises from variability associated with children, body mass index and gender [41]. Consequently, the secondary measure to estimate TBSAB will be the NSW Trauma Application and E-Burn Application. Available software programs have found some application but not much validation [42–44] and stems from the idea of rapid accurate TBSAB calculation to guide subsequent management. These mobile applications facilitate instant TBSAB calculation and both are used at the study centre and worldwide.

**Table 1:**
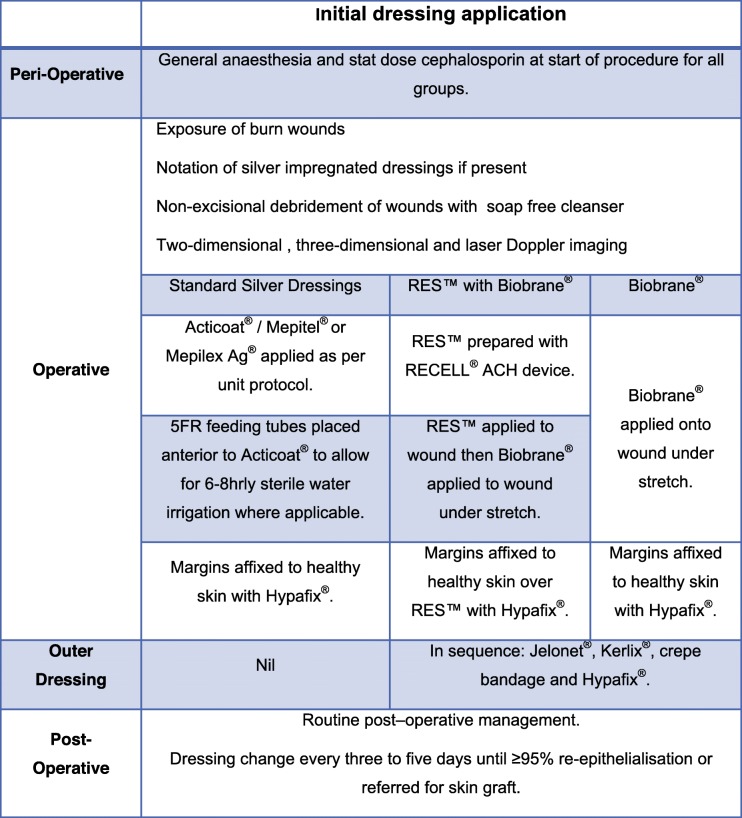
Initial dressing application procedure

*RES*^*TM*^ Regenerative Epidermal Suspension, *ACH* Autologous cell farvesting, *FR* French gauge

Objective burn depth will be measured with the Moor LDLS-BI Laser Doppler Imager® (Moor Instruments Ltd., Axminster, and Devon, UK). The reported sensitivity and specificity of Laser Doppler Imaging (LDI) in predicting outcome of paediatric injuries when compared with clinician assessment ranges from 80.6–97% and 76.9–100% [45–47], respectively. The assessment of burn depth by the attending burns surgeon will be taken as the primary approach. Burn depth assessed with LDI will be the secondary approach and measured at the initial dressing application after randomisation. Burn depth assessment using LDI in the first 48 h post-burn injury has been showed to be accurate [48–51] and is used to record burn depth progression and not guide clinical practice at the study site.

A three-part process will be required for the initial dressing application of participants in group B that includes harvesting of donor skin, preparation and application of the ASCS to the wound followed by Biobrane® and secondary dressings. A suitable donor site, where possible, will be identified adjacent to the wound. The size of skin sample will be based on the TBSAB as determined by the RES™ preparation guidelines. A donor sample of healthy skin will be obtained at a depth of 0.15 mm (0.006 in.) in depth using a pneumatic dermatome (Zimmer, Dover, Ohio, USA). The correct thickness of donor is evidenced by an almost translucent sample that leaves behind pinpoint bleeding at the donor site, the edges of the sample do not curl and there is an absence of the white dermis surface. Any excess RES™ if present will be applied to the donor site then dressed with Cuticerin**®** (Smith and Nephew Medical Ltd., Hull, UK). The sequence of secondary dressings used in groups B and C will maintain moisture, allow for absorption of exudate and a degree of compression to minimise disturbance of the fragile epidermal surface. Application of these dressing changes is not required under general anaesthesia unless otherwise indicated. The attending burns surgeon or paediatric surgery registrar assigned to the care of the patient will apply the RES™ and Biobrane®. Both doctors and nurses will apply the standard silver dressings.

### Post-operative care

Post-operatively, all in-patient participants will receive multi-disciplinary support as part of rehabilitation including allied health, multi-modal analgesia, and critical care for those hospitalised in the intensive care unit. Participants deemed fit to be discharged from the hospital in between the dressing changes prior to full re-epithelialisation; will be given a standardised patient information card with emergency numbers and basic dressing advice. In addition, participants will be advised to report any concerns regarding the dressing to staff at the study setting especially should fever or erythema develop at home. A schematic representation of the assessment timeline is illustrated in Table 2.

**Table 2:**
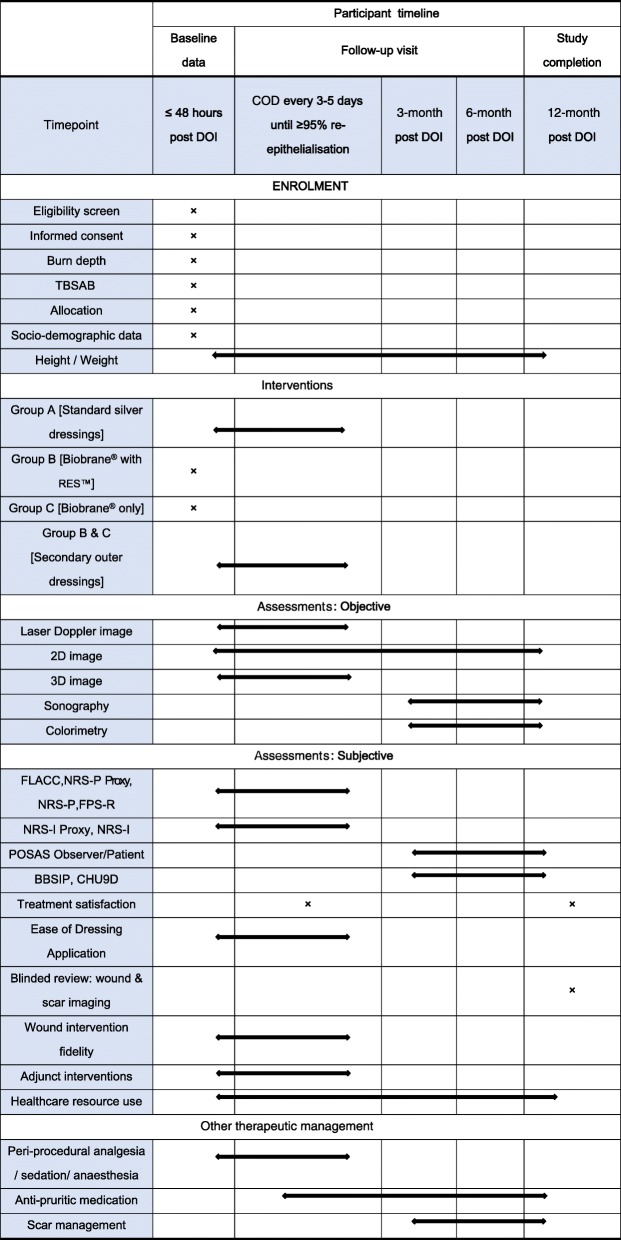
Data collection and assessment timeline for the trial

*COD* change of dressing, *DOI* date of injury, *TBSAB* total body surface area burned, *3D* three-dimensional, *2D* two-dimensional, *FLACC* Face, Legs, Activity, Cry, Consolability, *FPS-R* Faces Pain Scale-Revised, *NRS-P* Numeric Rating Scale–Pain, *NRS-P* *Proxy* Numeric Rating Scale–Pain Proxy, *NRS-I* Numeric Rating Scale–Itch, *NRS-I* *Proxy* Numeric Rating Scale–Itch Proxy, *POSAS* Patient and Observer Scar Assessment Scale, *BBSIP* Brisbane Burn Scar Impact Profile, *CHU9D* Child Health Utility 9D, *RES™* Regenerative Epithelial Suspension

### Monitoring

Minimal adverse events are expected from the proposed interventions. At the study centre, known potential adverse events (e.g. infection, haematoma, intensive care admissions) have a standardised management protocol. Adverse events related to the interventions will be monitored through review of patient medical records, by guardian and participant (where applicable) self-report and by the treating clinicians. All adverse events will be communicated to the clinical health service and Human Research Ethics Committee (HREC). Discontinuation or alteration of treatment will be at the discretion of the treating clinical team. An independent Safety Monitoring Group will also monitor all aspects of patient safety throughout the study on a regular basis. An interim analysis will be completed by an independent statistician, who will be blinded to the treatment allocation after a minimum of 30 participants have been recruited.

## Study outcomes

### Primary outcome: TTRE

Wound re-epithelialisation time is defined as the number of days from date of burn injury to ≥ 95% wound re-epithelialisation or date of skin graft as determined by the attending burns surgeon. Subjective assessment by an experienced burn surgeon is validated and reliable and will be the primary approach for TTRE [52, 53]. Objective assessment with three-dimensional (3D) digital camera imaging will be the secondary approach. Wound re-epithelialisaton will be expressed as a percentage using wound area (cm^2^) and perimeter (cm) calculated on the first dressing change day and the day a clinical assessment of ≥ 95% wound re-epithelialisation is made by the attending burns surgeon. The first dressing change is usually within 3 to 5 days after the burn injury. This allows for the burn wound to declare full extent and represent the maximal possible wound area in the absence of infection.

Images taken with the 3D LifeVizII System® (Quantificare S.A., Cedex, France) will be analysed using Dermapix® v3.0 (Quantificare S.A., Cedex, France). This fast, non-invasive 3D clinical imaging approach is ideal for children. The 3D LifeVizII System®/Dermapix® has been validated in the assessment of partial thickness burn injuries (intraclass correlation coefficient (ICC) 0.96, 95% confidence intervals (CI) 0.93–0.97) [54, 55]. However, the Dermapix® software is time-consuming as it requires the images to be first uploaded, then stitched and lastly, analysed. Hence, using Dermapix® does not provide a real-time analysis of the wound.

A second 3D camera, the Intel® RealSense™ Depth Camera D415 (Intel, CA, USA) with corresponding GPC 3D WoundCare (GPC, Swansea, UK) software will be used as a second secondary objective measure of TTRE. The reliability of the WoundCare Lite (ICC 0.985, 95% CI 0.905–0.996) [41] and instantaneous availability of analysed data output is advantageous over the 3D LifeVizII System®/Dermapix®. Validation in the paediatric burn wound population is currently in progress, as this technology is relatively new.

### Secondary outcome

Assessment of secondary outcomes will begin from initial dressing application and continue until the 12-month follow-up review as illustrated in Fig. 1 and Table 2.

### Acute pain

An 11-point numeric rating scale-pain (NRS-P) scores pain from 0 (no pain) to 10 (worst possible pain) [56, 57]. A proxy report of pain (NRS-P Proxy) by guardians of all participants will be the primary approach for acute pain. The Face, Legs, Activity, Cry and Consolability (FLACC) scale is an observational pain measure that has been validated for peri-procedural pain in children [58, 59] and scores pain intensity by rating five behaviours on a 3-point scale (0 to 2): face, legs, activity, consolability and cry. The revised Faces Pain Scale-Revised (FPS-R) utilises a horizontal scale of six facial expressions with assigned numeric values from 0 to 10 (0, 2, 4, 6, 8, 10), with 10 denoting maximal pain intensity [60]. Acute peri-procedural pain will be assessed subjectively by self-report from participants aged ≥ 8 years who are competent at seriation testing using the NRS-P and FPS-R pain scales. Behavioural observations will be made by both clinicians (FLACC scale) and guardian of all participants (NRS-P Proxy).

All sedation and analgesia administered at the dressing change will be documented. Pain assessments will be completed before and after dressing removal and application. This will be adhered to as much as is practically possible. In some circumstances, such as whilst under general anaesthesia, it will not be able to document participant self-report nor behavioural observations. Peri-procedural distraction techniques including bubbles, food, mobile device and toy that are part of standard care will also be recorded.

### Itch

Acute post-burn pruritis will be measured with an 11-point itch NRS-I (0 = no pruritis to 10 = worst imaginable pruritis) until the wound is ≥ 95% re-epithelialised. Participants ≥ 8 years will self-report (NRS-I). A proxy report of participant itch (NRS-I proxy) will be taken from the accompanying guardian for all participants and will the primary approach for itch. The NRS-I has shown good correlation when compared with visual analogue scale and Itch Man Scale and is easier to use in the evaluation of itch intensity [61, 62]. The itch item of the Patient and Observer Scar Assessment Scale (POSAS) will be used to assess chronic pruritis and will be evaluated by all participants 8 years or older as well as by all guardians (POSAS Patient) and by an investigator (POSAS Observer) [63].

### Scar severity

The re-epithelialised wound will be evaluated for the severity of scars using objective (ultrasonography and colorimetry) and subjective (POSAS and Brisbane Burn Scar Impact Profile (BBSIP)) measures [63–66]. The objective measures are the principal approach based on evidence of the ability to detect change. Scar thickness will be measured using the portable Venue40 MSK® Ultrasound machine (GE Healthcare, Fairfield, CT, USA). Ultrasound test-retest and interrater reproducibility of scar thickness is acceptable for paediatric scars [67].

Scar colorimetry will be evaluated with the DSM II ColorMeter® (Cortex Technology, Hadsund, Denmark). This device uses tristimulus reflectance, colorimetry and narrow-band photometry to assess scar lightness (L*), erythema (a*) and pigmentation (b*) [68]. An average of three measurements of L*, a*, b* from both the scar and healthy skin will be recorded. The primary measure of scar pigmentation and scar erythema will be L* and a* respectively.

Both the investigator and guardian of all participants will complete the POSAS. This survey assesses scar thickness, vascularity, pliability, pigmentation and relief, as well as patient scale items of itch, pain, colour, stiffness, thickness and irregularity [63]. The BBSIP physical scar subscales and BBSIP sensory subscale will also measure scar severity [64–66]. An investigator having completed scar assessment training to the satisfaction of the occupational therapist at the study site will complete the scar assessments of all participants.

### Health-related quality of life (HRQOL)

The BBSIP measures HRQOL and patient-reported scar characteristics for individuals with burn scars [64–66]. The Child Health Utility 9D (CHU9D) is a preference-based measure that assesses both participant HQROL and resource utilisation of the interventions [69–72]. Participant guardians will complete both HRQOL assessments for all participants.

### Treatment satisfaction

Treatment satisfaction is a patient-reported outcome [73] that guides quality of service delivery by a treating health service provider. In a study of parents (*n* = 62) of children receiving burn treatment, the perceived support and respectful communication by clinicians was strongly associated with the quality of received care [74]. Both clinicians and participant guardian will complete an 11-point NRS (0 = not at all satisfied to 10 = extremely satisfied) to measure treatment satisfaction.

### Ease of dressing application

The ease of dressing application will be assessed using a questionnaire regarding the dressings (application ease, dressing conformability, dressing application duration) including additional space for comments by treating clinicians.

### Healthcare resource use

Healthcare resource use related to the management of burn injuries will be collected for each patient from the perspective of the healthcare provider with a 12-month time-horizon. Healthcare resource use to be collected for each participant will include trial interventions and other wound management-related resource use. In addition, product(s) used in wound and scar management as well as associated clinician labour time (e.g. for assessments and treatments during outpatient clinic appointments). Resource use for burn-related hospitalisations (e.g. intensive care admission) will also be collected. Resource use will be costed at market rates (e.g. industrial award rates for clinician labour time, standing offer arrangement rates for products supplied, values consistent with the (Australian) Independent Hospital Pricing Authority for hospitalisation costs).

## Data management

### Sample size estimate

The sample size estimate conducted for this trial is based on the primary outcome of TTRE. Using the Power Analysis and Sample Size (PASS) software (version 11.0.7; PASS, NCSS, LLC), a two-sided logrank test procedure was used. A total sample size of 84 subjects (28 participants per group) that accounted for a 10% attrition rate would achieve 80% power at a significance level of 0.05 [10, 75, 76]. A clinically important difference of 4 days for re-epithelialisation was used for the sample size estimation.

### Randomisation and allocation

To reduce the chance of prediction of treatment allocation, a procedure which randomly allocates participants in groups using a random step size will be used [77]. The randomisation sequence will then be uploaded into Research Electronic Data Capture (REDCap) randomisation module [78] by a third party person not associated with the study. Upon obtaining informed consent for the participation, baseline data (participant demographics including Fitzpatrick Skin Type, burn injury time, depth, TBSAB, injury mechanism and history of first aid prior to enrollment) will be collected. An investigator aligned with the study will complete the randomisation using REDCap and inform the treating clinicians of the allocated intervention group.

### Blinding

Once data collection is complete, a panel of burn wound specialists inclusive of preselected burn surgeons and nurses will conduct a blinded review of wound and scar imaging. Any possible identifying material that could indicate to the blinded assessors, which group the participant, was allocated to, will be removed from images. The participants and their care providers will be blinded to their intervention assignment. Although it will be unavoidable to detect remnants of silver dressing in some cases, it is anticipated that it will be almost impossible to differentiate between the group B and group C. An experienced radiographer from the study centre will complete blinded assessments of scar thickness.

### Statistical analysis

In the exploratory stage, data will be graphed, and summary statistics will be calculated for all outcomes. Data will be analysed using the ‘Intention to Treat’ principle. The primary outcome data will use the survival analysis model (both Kaplan-Meier analysis and Cox proportional hazards regression), with the time to healing as the main outcome and dressing group as the explanatory variable. All other data will be analysed using appropriate methods for longitudinal data such as mixed model’s regression analysis or generalised estimating equations (GEE). A GEE is better suited for estimating population average effects whereas mixed models are better suited for understanding the source of correlation and its structure. Thus, they are complementary ways of analysing the data rather than opposing. It should also be noted that for symmetrical outcome variable distributions (such as normally distributed) both methods should give similar results. Statistical significance will be set at *p* < 0.05. The data set will be analysed with SPSS (IBM Corporation, Armonk, NY, USA) and Stata (StataCorp, College Station, TX, USA) software where appropriate.

### Data collection and storage

Data collection will be in the form of completion of questionnaires and clinical imaging (2D and 3D photographs, laser Doppler imaging and sonography), colorimetry, assessments of pain, itch intensity, treatment satisfaction, ease of dressing application, HRQOL, wound intervention fidelity, healthcare resource utilisation and scar severity as well as participant demographics, socioeconomic status and clinical characteristics. Data collection and management will be completed by an investigator and entered into the REDCap software [78] system for managing the data. At the study site, the de-identified data will be kept in a locked filing cabinet and backed up onto the Queensland University of Technology (QUT), Research Data Storage Service. This data will be stored for 15 years after the completion of the trial in accordance with National Health and Medical Research Council guidelines. Results of this study are to be published in peer-reviewed journals and presented at national and international burns conferences. This protocol was completed in accordance with the SPIRIT 2013 guidelines [79, 80].

## Discussion

The overall reported rate of hypertrophic scar formation in children ranges from 17 to > 50% in children [17]. To avoid the costly burden of managing burn scars and the impact on participants and families, current wound management approaches focus on shortening re-epithelialisation time. An ideal skin substitute provides effective and scar free healing. However, current wound management approaches incorporate some but not all properties of a functioning integumentary system thus the debate continues regarding how best to treat these burn wounds. For children with larger TBSAB, faster TTRE assists these burn survivors to begin the process of rehabilitation earlier, enables earlier return to routine daily activities and reduces scarring.

Effective pain management is of paramount importance, from both a psychological and clinical perspective in paediatric burn patients. Sub-optimal peri-procedural analgesia was significantly associated with a 2.2% delay in re-epithelialisation, for every point increase in pain measured using the FPS-R [81]. In addition, reducing the number of dressing changes may reduce the potential pain and distress associated with dressing changes. Post-burn pruritis causes much distress in children and has a moderate association with mental health [82]. Uncontrolled scratching hinders the re-epithelialisation process and potentially disrupts the fragile newly regenerated epithelium. In addition, the potential introduction of infective pathogens would also delay TTRE. Inevitably, a proportion of children post mixed partial thickness burn will develop a scar. A multi-disciplinary approach that incorporates both operative (contracture release, medical needling) and non-operative treatments (topical silicone, pressure garments) is vital to successful scar management. Efficient health resource utilisation improves the quality of health care provided to current and future children with mixed partial thickness burn injuries and most importantly enables clinicians to make informed decisions on best practice.

Based on past trials and studies at the study site, a limitation of this study is the anticipated large dropout rate. Potential attrition bias will be addressed by examining whether the extent and reasons for dropout are balanced across the groups [83]. Although different dropout rates across the three groups are not anticipated, a sensitivity analysis will be run to check for any differences. It is expected that random drop out will result in larger confidence intervals but not bias. In addition, if there is no imbalance, then attrition bias is not likely to be a problem [83]. Limitations associated with burn depth assessment and wound infection diagnosis will be adjusted for during statistical analysis. The BRACS randomised trial will add new knowledge regarding the comparative effectiveness of autologous skin cell suspension for the management of partial thickness and paediatric burn injuries.

## Trial status

Recruitment started in May 2018 and will proceed for a period of 18 months. Follow-up consultations will be in the outpatient setting at the 3, 6 and 12-month post-date of burn injury. Intended completion of the study is December 2020.

## Data Availability

Not applicable

## Abbreviations

2D: Two-dimensional
3D: Three-dimensional
ACH: Autologous cell harvesting
ASCS: Autologous skin cell suspension
BBSIP: Brisbane Burn Scar Impact Profile
BRACS: Biobrane®, RECELL® Autologous skin Cell suspension and Silver dressings
CHU9D: Child Health Utility 9D
COD: Change of dressing
DOI: Date of injury
FLACC: Face, Legs, Activity, Cry, Consolability
FPS-R: Faces Pain Scale-Revised
GEE: Generalised Estimating Equations
HREC: Human Research Ethics Committee
HRQOL: Health-related quality of life
ICC: Intraclass correlation coefficient
LDI: Laser Doppler Imaging
NRS-I Proxy: Numeric Rating Scale–Itch Proxy
NRS-I: Numeric Rating Scale–Itch
NRS-P Proxy: Numeric Rating Scale–Pain Proxy
NRS-P: Numeric Rating Scale–Pain
OPD: Outpatient department
POSAS: Patient and Observer Scar Assessment Scale
QLD: Queensland
QUT: Queensland University of Technology
REDCap: Research electronic data capture
RES™: Regenerative Epithelial Suspension
SPIRIT: Standard Protocol Items Recommendations for Interventional Trials
SSA: Site-specific approval
TBSAB: Total body surface area burned
TTRE: Time to re-epithelialisation

## References

[1] Peck MD (2012). Epidemiology and prevention of burns throughout the world. Jeschke MG, Kamolz L-P, Sjöberg F, Wolf SE, editors.

[2] Simons M, Price N, Kimble R, Tyack Z (2016). Patient experiences of burn scars in adults and children and development of a health-related quality of life conceptual model: a qualitative study. Burns..

[3] De Young AC, Kenardy JA, Cobham VE, Kimble R (2012). Prevalence, comorbidity and course of trauma reactions in young burn-injured children. J Child Psychol Psychiatry.

[4] Maskell J, Newcombe P, Martin G, Kimble R (2013). Psychosocial functioning differences in pediatric burn survivors compared with healthy norms. J Burn Care Res.

[5] Hop MJ, Polinder S, Vlies CH, Middelkoop E, Baar ME (2014). Costs of burn care: a systematic review. Wound Repair Regen.

[6] Tracy L, McInnes J, Gong J, Gabbe B, Thomas T (2017). BRANZ 8th Annual Report_ Jul 16 - Jun 17. Burns registry of Australia and New Zealand.

[7] Ter Horst B, Chouhan G, Moiemen NS, Grover LM (2018). Advances in keratinocyte delivery in burn wound care. Adv Drug Deliv Rev.

[8] Watt SM, Pleat JM (2018). Stem cells, niches and scaffolds: applications to burns and wound care. Adv Drug Deliv Rev.

[9] Gee Kee EL, Kimble RM, Cuttle L, Khan A, Stockton KA (2015). Randomized controlled trial of three burns dressings for partial thickness burns in children. Burns..

[10] Wood F, Martin L, Lewis D, Rawlins J, McWilliams T, Burrows S (2012). A prospective randomised clinical pilot study to compare the effectiveness of Biobrane(R) synthetic wound dressing, with or without autologous cell suspension, to the local standard treatment regimen in paediatric scald injuries. Burns..

[11] Mandal A (2007). Paediatric partial-thickness scald burns–is Biobrane the best treatment available?. Int Wound J.

[12] Lesher AP, Curry RH, Evans J, Smith VA, Fitzgerald MT, Cina RA (2011). Effectiveness of Biobrane for treatment of partial-thickness burns in children. J Pediatr Surg.

[13] Lonie S, Baker P, Teixeira RP (2017). Healing time and incidence of hypertrophic scarring in paediatric scalds. Burns..

[14] Gee Kee E, Stockton K, Kimble RM, Cuttle L, McPhail SM (2017). Cost-effectiveness of silver dressings for paediatric partial thickness burns: an economic evaluation from a randomized controlled trial. Burns..

[15] Deitch EA, Wheelahan TM, Rose MP, Clothier J, Cotter J (1983). Hypertrophic burn scars: analysis of variables. J Trauma.

[16] Cubison TC, Pape SA, Parkhouse N (2006). Evidence for the link between healing time and the development of hypertrophic scars (HTS) in paediatric burns due to scald injury. Burns..

[17] Chipp E, Charles L, Thomas C, Whiting K, Moiemen N, Wilson Y (2017). A prospective study of time to healing and hypertrophic scarring in paediatric burns: every day counts. Burns Trauma.

[18] Ketchum LD (1977). Hypertrophic scars and keloids. Clin Plast Surg.

[19] Vloemans AF, Hermans MH, van der Wal MB, Liebregts J, Middelkoop E (2014). Optimal treatment of partial thickness burns in children: a systematic review. Burns..

[20] Liu Hai-Fei, Zhang Feng, Lineaweaver William C. (2017). History and Advancement of Burn Treatments. Annals of Plastic Surgery.

[21] Cuttle L, Naidu S, Mill J, Hoskins W, Das K, Kimble RM (2007). A retrospective cohort study of Acticoat versus Silvazine in a paediatric population. Burns..

[22] Khundkar R, Malic C, Burge T (2010). Use of Acticoat dressings in burns: what is the evidence?. Burns..

[23] Hajska M, Dragunova J, Koller J (2017). Cytotoxicity testing of burn wound dressings: first results. Cell Tissue Bank.

[24] White R, Morris C (2009). Mepitel: a non-adherent wound dressing with Safetac technology. Br J Nurs.

[25] Silverstein P, Heimbach D, Meites H, Latenser B, Mozingo D, Mullins F (2011). An open, parallel, randomized, comparative, multicenter study to evaluate the cost-effectiveness, performance, tolerance, and safety of a silver-containing soft silicone foam dressing (intervention) vs silver sulfadiazine cream. J Burn Care Res.

[26] Zhao H, Chen Y, Zhang C, Fu X (2016). Autologous epidermal cell suspension: a promising treatment for chronic wounds. J Tissue Viability.

[27] Wood F (2003). Clinical potential of autologous epithelial suspension. Wounds.

[28] Fan C, Pek CH, Por YC, Lim GJS (2018). Biobrane dressing for paediatric burns in Singapore: a retrospective review. Singap Med J.

[29] Gravante G, Di Fede MC, Araco A, Grimaldi M, De Angelis B, Arpino A (2007). A randomized trial comparing ReCell system of epidermal cells delivery versus classic skin grafts for the treatment of deep partial thickness burns. Burns..

[30] Holmes Iv JH, Molnar JA, Carter JE, Hwang J, Cairns BA, King BT (2018). A comparative study of the ReCell(R) device and autologous spit-thickness meshed skin graft in the treatment of acute burn injuries. J Burn Care Res.

[31] Dunne JA, Rawlins JM (2014). Early paediatric scald surgery—a cost effective dermal preserving surgical protocol for all childhood scalds. Burns..

[32] Austin RE, Merchant N, Shahrokhi S, Jeschke MG (2015). A comparison of Biobrane and cadaveric allograft for temporizing the acute burn wound: cost and procedural time. Burns..

[33] Greenwood JE, Clausen J, Kavanagh S (2009). Experience with biobrane: uses and caveats for success. Eplasty..

[34] Hyland EJ, D'Cruz R, Menon S, Harvey JG, La Hei E, Lawrence T (2018). Biobrane (TM) versus acticoat (TM) for the treatment of mid-dermal pediatric burns: a prospective randomized controlled pilot study. Int J Burns Trauma.

[35] Haddad AG, Giatsidis G, Orgill DP, Halvorson EG (2017). Skin substitutes and bioscaffolds: temporary and permanent coverage. Clin Plast Surg.

[36] Woodruff E (1984). Biobrane, a Biosynthetic Skin Prosthesis in Burn Wound Coverings. DL W, editor.

[37] Demling RH (1995). Use of Biobrane in the management of scalds. J Burn Care Rehabil.

[38] Phillips LG, Robson MC, Smith DJ, Phillips WA, Gracia WD, McHugh TP (1989). Uses and abuses of a biosynthetic dressing for partial skin thickness burns. Burns..

[39] Lal S, Barrow RE, Wolf SE, Chinkes DL, Hart DW, Heggers JP (2000). Biobrane® improves wound healing in burned children without increased risk of infection. Shock..

[40] Government Q (2018). Queensland population counter: The State of Queensland (Queensland Treasury).

[41] Farrar E, Pujji O, Jeffery S (2017). Three-dimensional wound mapping software compared to expert opinion in determining wound area. Burns..

[42] Fontaine M, Ravat F, Latarjet J (2018). The e-burn application - a simple mobile tool to assess TBSA of burn wounds. Burns..

[43] Management NIoTaI (2015). NSW Trauma App Analysis Report 21st August - 8th November 2015. Institute of Trauma and Injury Management.

[44] Management NIoTaI (2016). NSW Trauma App Analysis Report August 2015 – August 2016. NSW Institute of Trauma and Injury Management.

[45] Holland AJA, Martin HCO, Cass DT (2002). Laser Doppler imaging prediction of burn wound outcome in children. Burns..

[46] La Hei ER, Holland AJ, Martin HC (2006). Laser Doppler imaging of paediatric burns: burn wound outcome can be predicted independent of clinical examination. Burns..

[47] Cho JK, Moon DJ, Kim SG, Lee HG, Chung SP, Yoon CJ (2009). Relationship between healing time and mean perfusion units of laser Doppler imaging (LDI) in pediatric burns. Burns..

[48] Mileski WJ, Atiles L, Purdue G, Kagan R, Saffle JR, Herndon DN (2003). Serial measurements increase the accuracy of laser Doppler assessment of burn wounds. J Burn Care Rehabil.

[49] Jeng JC, Bridgeman A, Shivnan L, Thornton PM, Alam H, Clarke TJ (2003). Laser Doppler imaging determines need for excision and grafting in advance of clinical judgment: a prospective blinded trial. Burns..

[50] Mill J, Cuttle L, Harkin DG, Kravchuk O, Kimble RM (2009). Laser Doppler imaging in a paediatric burns population. Burns..

[51] Wang XQ, Mill J, Kravchuk O, Kimble RM (2010). Ultrasound assessed thickness of burn scars in association with laser Doppler imaging determined depth of burns in paediatric patients. Burns..

[52] Bloemen M, Boekema B, Vlig M, van Zuijlen P, Middelkoop E (2012). Digital image analysis versus clinical assessment of wound epithelialization: a validation study. Burns..

[53] Bloemen MC, van Zuijlen PP, Middelkoop E (2011). Reliability of subjective wound assessment. Burns..

[54] Stockton KA, McMillan CM, Storey KJ, David MC, Kimble RM (2015). 3D photography is as accurate as digital planimetry tracing in determining burn wound area. Burns..

[55] Gee Kee EL, Kimble RM, Stockton KA (2015). 3D photography is a reliable burn wound area assessment tool compared to digital planimetry in very young children. Burns..

[56] Page MG, Katz J, Stinson J, Isaac L, Martin-Pichora AL, Campbell F (2012). Validation of the numerical rating scale for pain intensity and unpleasantness in pediatric acute postoperative pain: sensitivity to change over time. J Pain.

[57] von Baeyer CL, Spagrud LJ, McCormick JC, Choo E, Neville K, Connelly MA (2009). Three new datasets supporting use of the numerical rating scale (NRS-11) for children’s self-reports of pain intensity. Pain..

[58] Merkel S, Voepel-Lewis T, Shayevitz J, Malviya S (1994). FLACC Pain Assessment Tool: Reliability and Validation with Existing Tools. Anesthesiology.

[59] von Baeyer CL, Spagrud LJ (2007). Systematic review of observational (behavioral) measures of pain for children and adolescents aged 3 to 18 years. Pain..

[60] Huguet A, Stinson JN, McGrath PJ (2010). Measurement of self-reported pain intensity in children and adolescents. J Psychosom Res.

[61] Reich A, Halupczok J, Ramus M, Stander S, Szepietowski J (2011). New Data on the Validation of Vas and Nrs in Pruritus Assessment: Minimal Clinically Important Difference and Itch Frequency Measurement. Acta Derm Venereol.

[62] Nieuwendijk SMP, de Korte IJ, Pursad MM, van Dijk M, Rode H (2018). Post burn pruritus in pediatric burn patients. Burns..

[63] Draaijers LJ (2003). The patient and observer scar assessment scale: a reliable and feasible tool for scar evaluation. Plast Reconstr Surg.

[64] Tyack Z, Kimble R, McPhail S, Plaza A, Simons M (2017). Psychometric properties of the Brisbane burn scar impact profile in adults with burn scars. PLoS One.

[65] Tyack Z, Ziviani J, Kimble R, Plaza A, Jones A, Cuttle L (2015). Measuring the impact of burn scarring on health-related quality of life: development and preliminary content validation of the Brisbane burn scar impact profile (BBSIP) for children and adults. Burns..

[66] Simons M, Kimble R, McPhail S, Tyack Z. The longitudinal validity, reproducibility and responsiveness of the Brisbane burn scar impact profile (caregiver report for young children version) for measuring health-related quality of life in children with burn scars. Burns. 2019. *Article in Press*. 10.1016/j.burns.2019.04.015 31147101

[67] Simons M, Kee EG, Kimble R, Tyack Z (2017). Ultrasound is a reproducible and valid tool for measuring scar height in children with burn scars: a cross-sectional study of the psychometric properties and utility of the ultrasound and 3D camera. Burns..

[68] Draaijers LJ, Tempelman FR, Botman YA, Kreis RW, Middelkoop E, van Zuijlen PP (2004). Colour evaluation in scars: tristimulus colorimeter, narrow-band simple reflectance meter or subjective evaluation?. Burns..

[69] Stevens KJ (2010). Working with children to develop dimensions for a preference-based, generic, pediatric health-related quality-of-life measure. Qual Health Res.

[70] Stevens KJ (2009). Developing a descriptive system for a new preference-based measure of health-related quality of life for children. Qual Life Res.

[71] Stevens KJ (2011). Assessing the performance of a new generic measure of health related quality of life for children and refining it for use in health state valuation. Appl Health Econ Health Policy.

[72] Stevens K (2010). The Child Health Utility 9D (CHU9D) – A New Paediatric Preference Based Measure of Health Related Quality of Life. PRO Newsletter.

[73] Revicki DA (2004). Patient assessment of treatment satisfaction: methods and practical issues. Gut.

[74] Willebrand M, Sjöberg F, Huss F, Sveen J (2018). Parents’ perceived quality of pediatric burn care. J Crit Care.

[75] Lakatos E (1988). Sample Sizes Based on the Log-Rank Statistic in Complex Clinical Trials. Biometrics.

[76] Lakatos E (2002). Designing Complex Group Sequential Survival Trials. Stat Med.

[77] Snow G (2013). blockrand: Randomization for block random clinical trials.

[78] Harris PA, Taylor R, Thielke R, Payne J, Gonzalez N, Conde JG (2009). Research electronic data capture (REDCap) – a metadata-driven methodology and workflow process for providing translational research informatics support. J Biomed Inform.

[79] Chan AW, Tetzlaff JM, Altman DG, Laupacis A, Gotzsche PC, Krleza-Jeric K (2013). SPIRIT 2013 statement: defining standard protocol items for clinical trials. Ann Intern Med.

[80] Chan AW, Tetzlaff JM, Gotzsche PC, Altman DG, Mann H, Berlin JA (2013). SPIRIT 2013 explanation and elaboration: guidance for protocols of clinical trials. BMJ..

[81] Brown NJ, Kimble RM, Gramotnev G, Rodger S, Cuttle L (2014). Predictors of re-epithelialization in pediatric burn. Burns..

[82] McGarry S, Burrows S, Ashoorian T, Pallathil T, Ong K, Edgar DW (2016). Mental health and itch in burns patients: potential associations. Burns..

[83] Moher D, Hopewell S, Schulz KF, Montori V, Gøtzsche PC, Devereaux PJ (2010). CONSORT 2010 explanation and elaboration: updated guidelines for reporting parallel group randomised trials. J Clin Epidemiol.

